# State of the Art: The Immunomodulatory Role of MSCs for Osteoarthritis

**DOI:** 10.3390/ijms23031618

**Published:** 2022-01-30

**Authors:** Dae Gyu Kwon, Myung Ku Kim, Yoon Sang Jeon, Yoon Cheol Nam, Jin Seong Park, Dong Jin Ryu

**Affiliations:** Orthopedic Surgery, Inha University Hospital, 22332 Inhang-ro 27, Jung-gu, Incheon 22332, Korea; gundegy77@gmail.com (D.G.K.); m9kim@inha.ac.kr (M.K.K.); ysjeon80@hanmail.net (Y.S.J.); cheolz@hanmail.net (Y.C.N.); dhdnfkdl@gmail.com (J.S.P.)

**Keywords:** mesenchymal stem cell, antiinflammation, immunomodulation, osteoarthritis, exosome, miRNA

## Abstract

Osteoarthritis (OA) has generally been introduced as a degenerative disease; however, it has recently been understood as a low-grade chronic inflammatory process that could promote symptoms and accelerate the progression of OA. Current treatment strategies, including corticosteroid injections, have no impact on the OA disease progression. Mesenchymal stem cells (MSCs) based therapy seem to be in the spotlight as a disease-modifying treatment because this strategy provides enlarged anti-inflammatory and chondroprotective effects. Currently, bone marrow, adipose derived, synovium-derived, and Wharton’s jelly-derived MSCs are the most widely used types of MSCs in the cartilage engineering. MSCs exert immunomodulatory, immunosuppressive, antiapoptotic, and chondrogenic effects mainly by paracrine effect. Because MSCs disappear from the tissue quickly after administration, recently, MSCs-derived exosomes received the focus for the next-generation treatment strategy for OA. MSCs-derived exosomes contain a variety of miRNAs. Exosomal miRNAs have a critical role in cartilage regeneration by immunomodulatory function such as promoting chondrocyte proliferation, matrix secretion, and subsiding inflammation. In the future, a personalized exosome can be packaged with ideal miRNA and proteins for chondrogenesis by enriching techniques. In addition, the target specific exosomes could be a gamechanger for OA. However, we should consider the off-target side effects due to multiple gene targets of miRNA.

## 1. Introduction

Osteoarthritis (OA) has a multifactorial etiology, including aging, obesity, previous injury, female, hormone level, and epigenetics [1,2,3]. The articular cartilages receive their nutrients by synovial fluid infiltration, which make it difficult for the cartilage to repair after injury [4,5]. The chondrocyte is the primary cell type in cartilage tissue, making up only 5–10% of the total mass [6]. Chondrocytes are confined within the extracellular matrix (ECM), which possibly limits their ability to migrate to injured areas [7]. Thus, the damaged cartilage has limited self-regenerative potential and is finally replaced by fibrocartilage or scar tissue with poorer functional and structural properties. [8]. The molecular mechanism of OA pathogenesis is not fully understood; however, both inflammation and chondrocytes are considered to play important roles [1]. Especially, a low-grade, chronic inflammatory reaction contributes to disease progression. Besides inflammatory cascades, biomechanical and oxidative stress compromises the viability of chondrocytes, leading to catabolic process with further ECM degradation [9].

The goal of treatment for early- and mid-stage OA is to prevent further damage, achieve symptoms control, and return to normal joint cartilage conditions. Nonsteroidal anti-inflammatory drugs (NSAIDs) medication and corticosteroid injections are often used for many years; however, these strategies have no impact on the progressive degeneration of joint tissues [10,11]. Recent studies suggested the possibility of disease modifying osteoarthritis drugs (DMOAD); however, there is no officially recognized therapeutic agents yet [12,13]. Although some inflammatory mediators targeting therapies such as IL-1, IL-6, or TNF-a have been evaluated, however, they resulted in a majority of disappointing results [14,15,16].

Mesenchymal stromal/stem cells (MSCs)-based therapy seems to be in the spotlight because this strategy could provide an enlarged anti-inflammatory and regenerative potential. The regenerative effects of MSCs are mediated by their paracrine effects with chondroprotective and anti-inflammatory functions [17,18]. In addition, owing to the capacity of MSCs for self-renewal, multi-differentiation, and immunoregulatory function, MSC-based therapy has excellent potential for cartilage regeneration. [19]. Although the clinical results so far with the intra-articular injection of MSCs to induce cartilage regeneration have been disappointing, MSCs implantation using various surgical techniques with scaffolds is being increasingly attempted. Currently, the widely used method is microfracture, autologous or allogenic cartilage transplantation, and autologous chondrocyte implantation; however, these treatment methods require surgery or have several limitations [20,21,22,23,24]. Recently, MSCs-derived exosomes have just been tried in OA therapy [18,25,26]. In this review, we discuss the current status of MSCs therapies, focusing on the immunomodulatory effects for OA.

## 2. The Effect of Inflammation in OA Progression

**Figure 1:**
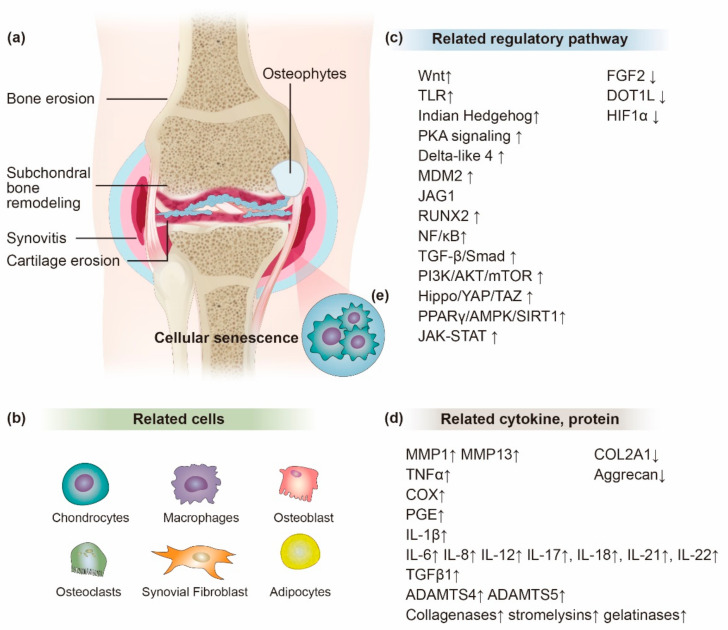
The “inflammatory” pathogenesis of osteoarthritis (OA). (**a**) OA is not only damage of cartilage, but also surrounding joint tissue including inflammation of the synovium, subchondral bone remodeling, bone erosion, and osteophyte formation were accompanied. (**b**) In addition, cells in the affected joint tissues actively participate in the OA initiation and progression. (**c**) Lots of regulatory pathways are related to OA onset and progression; however, not all are necessarily implicated in all phenotypes of the OA progression. (**d**) During OA progression, multiple inflammatory cytokines and proteins are involved in damaging cartilage and promote an endless cycle of inflammation. (**e**) Senescent chondrocytes themselves also trigger an inflammatory response to the surrounding area.

Although OA has been introduced as a degenerative disease, currently, OA is understood as a low-grade chronic inflammatory process that could promote disease symptoms and accelerate disease progression [27,28]. Catabolic and proinflammatory factors are produced by the damaged chondrocyte and inflamed synovium and alter the balance of cartilage matrix anabolism and catabolism, leading to the production of redundant proteolytic enzymes, giving rise to cartilage breakdown [29,30]. The changes in cartilage and subchondral bone cause further synovitis, resulting in a vicious cycle. Progressive synovitis aggravates clinical symptoms and stimulates further joint degeneration (Figure 1) [31].

Chondrocytes are spatially isolated by a large volume of ECM and are responsible for the synthesis and maintenance of the ECM [32]. The framework of ECM includes collagens (mainly type II collagen), proteoglycans (mainly aggrecan), and several bioactive factors. The supply of chondrocyte nutrients and the disposal of metabolic waste occur through the ECM [33]. The activity of chondrocytes, including their response to stimuli, controls the synthesis of new ECM components, a process influenced by aging [34,35]. The ability of cartilage repair declines, manifested by a decline in chondrocyte number leading to age-associated changes in ECM composition [34,36]. These changes result in degeneration of the cartilage and limit its ability of repair [37]. In recent years, accumulating evidence has suggested that OA should be considered a disease of the whole joint [38]. Articular cartilage and subchondral bone form an integral unit that undergoes uncontrolled catabolic and anabolic remodeling during OA development [39,40].

Senescence of chondrocyte also affects the contribution to the pathogenesis of OA. The proportion of senescent cells in joints is strongly related to age [41,42]. Senescent cells revealed a distinct senescence-associated secretory phenotype (SASP). SASP is expressed by the overproduction of proteolytic and proinflammatory factors and reactive oxygen species (ROS) generation, which are harmful to the surrounding joint tissue, consequently joint destruction [43]. Premature chondrocyte senescence can be induced by repeated mechanical stress, traumatic injury, obesity, and finally leading to early OA [43,44].

Cytokines secreted by the immune cells are the key players of arthritic changes [45]. Proinflammatory cytokines, such as IL-1β and TNF-α, are secreted in early OA and actively drive the inflammatory pathway independently or in collaboration with other cytokines [46,47]. Especially, IL-1β is one of the critical mediators of cartilage destruction in OA [48,49]. IL-1β are produced by injured synoviocytes, chondrocytes, and mononuclear cells [31]. Upon inflammatory stimulation, the cells release IL-1β, IL-6, IL-8, TNF-α, and A Disintegrin and Metalloproteinase with Thrombospondin motifs (ADAMTS) [49,50,51,52,53,54]. OA joint showed that IL-1β, IL-6, IL-8, IL-17, IL-18, IL-22, and transforming growth factor-beta 1 (TGF-β1) were increased in the inflamed joint tissues compared to the noninflamed tissues [9]. A similar cytokine profile pattern was observed in OA animal models [55].

Moreover, IL-1β affects matrix metallopeptidase (MMP)s’ synthesis by chondrocytes, including MMP-1 and MMP-13, which destroy the articular cartilage [56]. MMPs are a class of proteinases responsible for the degradation of collagen-II and proteoglycans in the articular cartilage. MMPs play vital roles in ECM degradation in OA. Especially, MMP-13 is an important member of proteinases [57,58]. In addition, IL-1β was shown to induce the production of ROS such as nitric oxide (NO) [59]. IL-1β stimulates expression of TNF-α and expression of TNF receptor (TNFR) in chondrocytes. The binding of TNF-α to TNFR causes signal transduction and activates TNF receptor-associated factor2 (TRAF2). TRAF2 activates the nuclear factor kappa-light-chain-enhancer of activated B cell (NF-κB) signaling pathway involved in inflammatory diseases [60]. IL-17 induces the release of IL-6, IL-8, and TNF-α by synovial fibroblasts and chondrocytes, leading to inflammation and cartilage breakdown [61]. IL-17 also promotes the recruitment and activation of neutrophils, which are the initial cell types recruited to the inflammation sites [62].

The inflammatory process activates the release of enzymes by damaged chondrocytes and monocytes resulting in the enhanced catabolic process [50]. These enzymes include proteins of ADAMTS family and MMP-1, 3, 13, which are directly responsible of ECM remodeling. Alarmins (high-mobility group box protein 1 S100A8 and S100A9) by monocytes also contribute to the inflammatory cascade [63]. MMP-13, ADAMTS-4, and ADAMTS-5 are used as catabolic markers, while COL2A1 and ACAN are used as the anabolic markers for cartilage metabolism [50,64].

Several signaling pathways have been implicated in OA development and progression. These pathways include Wnt/β-catenin, DOT1L, PI3K/Akt/mTOR, SIRIT/AMPK, Hippo- YAP/TAZ, NF-κB, NLRP3 inflammasome-mediated pyroptosis, and HIF-1-VEGF-Notch pathways [1,65,66,67]. Another crucial pathway related in the inflammatory progression of OA is the mitogen-activated protein kinases (MAPK) pathway, which comprises the extracellular signal-regulated kinase 1/2 (ERK1/2), the c-Jun N-terminal kinase (JNK), p38, and ERK5 cascades [68]. In addition, a number of microRNA (miRNAs) and long noncoding RNA (lncRNAs) have also been identified to mediate OA pathogenesis [69]. Moreover, the activation of the innate immune system also contributes to the persistence of synovial low-grade inflammation. The damage to cartilage and ECM resulting from repeated senescence and microtrauma generates damage-associated molecular patterns (DAMPs) which activate the innate immune system through the toll-like receptor (TLR) pathway [28].

Although lots of signaling pathways and the role of cytokines have been evaluated, OA subtypes exhibit variable pathologic pathways, making the development of effective therapies challenging [44].

## 3. The Mesenchymal Stem Cells

MSCs, a precursor of connective tissue cells, can be isolated from a variety of adult or neonatal tissues [39]. MSCs are pluripotent progenitor cells that possess self-renewal capability and can differentiate into multiple lineages including adipocytes, osteoblasts, and chondrocytes [70]. They are characterized by their fibroblastic shape and their immunophenotype (CD11b^-^, CD14^-^, CD34^-^, CD45^-^, HLA-DR^-^, CD73^+^, CD90^+^, and CD105^+^) [9,71]. Among the various MSCs subpopulations, CD49f^+^, CD146^+^, CD105^+^, CD271^+^, and Stro-1^+^ MSCs showed great potential for improvement of cartilage repair. [72]. In some MSCs, subpopulations have been shown to have a significantly vital ability for proliferation, migration, immunomodulation, or chondrogenesis and have great potential for the applying of MSC-based cartilage regeneration strategies [73].

MSCs release some immunomodulatory factors and express various cytokine and chemokine receptors, which enable migration to injury and inflammation sites [74]. Although, MSCs disappear from the delivered tissue quickly after administration but are still able to deliver chondroprotective and immunomodulatory effect [75]. MSCs promote cartilage regeneration by modulating the host environment and stimulating the endogenous progenitors. As a paradigm-shifting from replacement to tissue regeneration, MSCs therapy was tried for many orthopedic diseases, especially for OA [76].

### 3.1. Source of MSCs

Currently, bone marrow stem cell (BMSCs), adipose-derived mesenchymal stem cells (ADSCs), synovium-derived mesenchymal stem cells (SDMSCs), and human umbilical cord blood-derived mesenchymal stem cells/Wharton’s jelly-derived mesenchymal stem cells (hUCBDMSCs/WJDMSCs) are the most widely used sources of MSCs in cartilage tissue regeneration, each with its respective characteristic advantages for cartilage regeneration. However, there is heterogeneity in their regeneration potential for cartilage repair, including their accessibility, immunogenicity, donor site morbidity, and chondrogenic, proliferative, and immunomodulatory ability [72].

### 3.2. Bone Marrow-Derived Mesenchymal Stem Cells (BMSCs)

MSCs isolated from autologous bone marrow have been widely used in the clinical field to investigate their chondrogenic potential for OA treatment. The safety and effectiveness for immunomodulatory functions of BMSCs have been reported for many years [21,23,77]. After being activated by inflammatory factors, BMSCs are able to secrete indoleamine 2,3-dioxygenase (IDO) and promote M2 macrophage polarization, and these macrophages tend to secrete more IL-10 and less IL-1β. This process resulted in better chondrocyte survival time in vivo [78]. Seeding BMSCs on polyglycolic acid/polylactic acid scaffolds can induce chondrogenesis and construct mature cartilage in vitro [79].

Autologous BMSCs can reduce joint synovial inflammation, as evidenced by decreased levels of proinflammatory macrophages, monocytes, and IL-12 [80]. Vega and colleagues attempted to inject an allogenic BMSCs and found that its therapeutic effect was significantly higher than that of hyaluronic acid, with no definite adverse reaction [81]. Nevertheless, newly formed cartilage by injecting BMSCs is often structurally uneven. Biomaterials can solve this problem by providing a framework or scaffold for regenerated tissue [72].

### 3.3. Adipose Tissue-Derived Mesenchymal Stem Cells (ADMSCs)

Because of easy accessibility to obtain large number of MSCs, ADMSCs represent a more easily available source of MSCs for cartilage repair [82]. Compared with BMSCs, ADMSCs are more easily cultured and grow more rapidly [83]. The main benefits of ADMSCs are their ease of potential for isolation and manipulability, and, furthermore, their telomerases are less affected by age than BMSCs [84]. Adipose tissue is usually acquired from subcutaneous fat of the abdomen or inner thigh area, while subpatellar fat tissue obtained by arthroscopy procedure could be another source for ADSCs.

Although many studies have reported that the chondrogenicity of ADSCs is inferior to that of BMSCs; however, ADSCs reveal more immunoregulatory potential than BMSCs, as evidenced by greater IDO activity [85,86]. ADSCs can induce the transition of immune cells toward an anti-inflammatory phenotype in the joint. Interestingly, low-dose (2 × 10^6^ cells) ADSCs transplantation improved the pain score and the knee function score for severe OA patients more than high-dose (5 × 10^7^) ADSCs transplantation [17,87].

### 3.4. Synovial Membrane-Derived Mesenchymal Stem Cells (SDMSCs)

SDMSCs display greater chondrification, greater proliferation, and chondrogenic differentiation potential than BMSCs and ADMSCs [88,89]. Cocultured SDSCs with chondrocytes showed higher COL2A1 and Sox9 expression, which suggest that the coculture of SDSCs and chondrocytes could promote ECM deposition and inhibit the osteogenic differentiation of chondrocytes [90].

However, for immunomodulatory capacity, compared with BMSC, the expression of HLA-DR on SDMSCs was significantly reduced, suggesting that its potential immunogenicity was reduced [90]. Although only a few clinical studies reported good outcomes of SDSCs, the studies of SDSCs are not as enough as those on BMSCs and ADSCs. For clinical use of SDSCs for cartilage regeneration, we need more clinical trials and more advanced biomaterials to enhance the regenerative ability [91].

### 3.5. Human Umbilical Cord Blood/Wharton’s Jelly-Derived Mesenchymal Stem Cells (WJDMSCs)

The mucoid Wharton’s jelly also known as intervascular umbilical cord tissue is composed of fibroblast-like cells recognized as pluripotent MSCs capable of differentiating into chondrocytes in vitro and in vivo [24,92]. WJDMSCs have been reported to have a great potential for proliferation and chondrogenic differentiation than BMSCs [93]. Intra-articular injection of WJDMSCs decreased expression of the proinflammatory cytokines and MMPs in the synoviocytes [94]. More importantly, WJDMSCs are characterized by low immunogenicity and excellent immunoregulatory ability, and these characteristics are maintained even after their differentiation into mature phenotypes [95,96]. Furthermore, there was no obvious immune rejection when WJDMSCs were subcutaneously transplanted into rats. Thus, the immune properties of WJDMSCs make them a great source for repairing damaged cartilage [94,97].

Beyond the advantages of strong proliferation and differentiation, WJDMSCs have a uniform immunophenotype, noninvasive acquisition procedures, and no ethical controversy [96]. Furthermore, suspensions of MSCs sourced from Wharton’s jelly may be stored for long periods while maintaining cell viability, allowing for off-the-shelf use [98]. However, the storage and transportation conditions of the cells are stringent. Although the probability is known to be very low [95], the risk of disease transmission, tumorigenicity, and possible immune rejection of MSCs hinder the application of tissue-engineered cartilage based on MSCs [96].

## 4. Mode of Anti-Inflammatory and Immunomodulatory Actions of MSCs for OA

**Figure 2:**
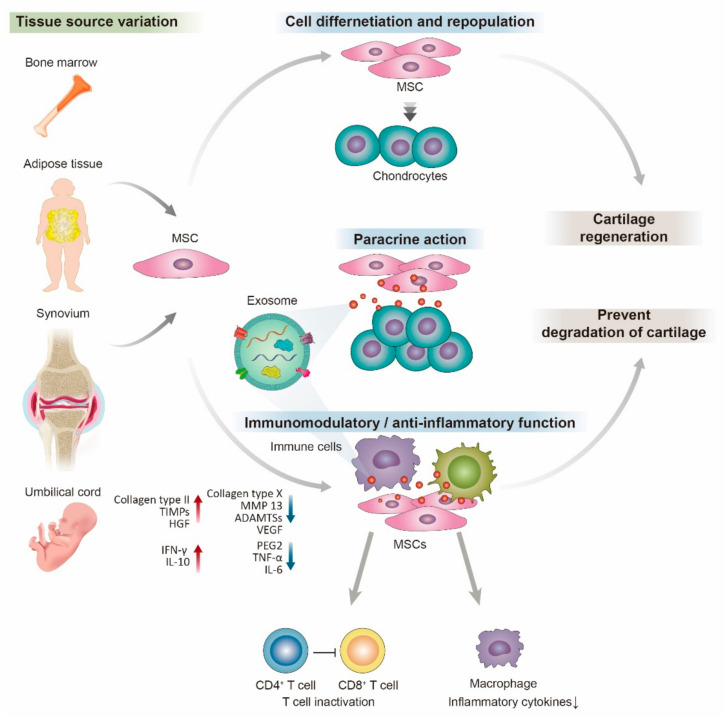
Schematic presentation of the mesenchymal stem cells (MSCs) in cartilage regeneration. MSCs can be recruited from bone marrow, adipose tissue, synovium, and umbilical cord blood. MSCs induce cartilage regeneration by various mechanisms. MSCs can proliferate and differentiate directly into chondrocytes to replace damaged cells. In addition, MSCs can secrete exosomes including cytokines and miRNA to maintain chondrocyte phenotypes and promote their proliferation and ECM composition as a paracrine effect. Furthermore, MSCs can exert immunomodulatory and anti-inflammatory functions on numerous immune cells through exosome secretion. The exosome signals prohibit the inflammatory pathway, prevent cartilage degradation, and promote cartilage regeneration.

The therapeutic efficacy of MSCs is considered to mainly be by paracrine effect and seems to be independent of their engraftment. MSCs revealed different functions due to a variety of secreted factors. They produce growth factors, such as TGF-β, vascular endothelial growth factor (VEGF), basic fibroblast growth factor (FGF), or hepatocyte growth factor (HGF), which induces proliferation and angiogenesis of various cell types [9,99]. Another important role of MSCs is to rescue target cells from apoptosis induced by trauma, oxidative environment. Some key proteins have been suggested to play such a role. Insulin growth factor (IGF)-1, IL-6, and stanniocalcin-1 are essential for reversal apoptosis in fibroblasts, while VEGF, HGF, and TGF-β1 have been shown to protect against apoptosis (Figure 2) [100].

In addition, after exposure to injured tissue or inflammatory cytokines, MSCs can exert immunomodulatory and immunosuppressive effects on various immune cells [72]. MSCs mediated the immunomodulatory function by programmed death-ligand 1 (PD-L1) and Fas ligand (FasL) [101]. MSCs interact with T cells and inhibit the proliferation and differentiation of native T lymphocytes toward the Th1 or Th17 phenotype [102]. MSCs also can control the repolarization of Th17 cells through PD-L1 expression. The inhibition of differentiation of naive T lymphocytes was related to increases in the number of functional natural Treg cells and enhanced IL-10 secretion [103]. The presence of IL-17A, MSCs showed more PGE2 and markedly increased the proportion of CD4+Foxp3+ Tregs and suppressed T-cell proliferation [104]. IL-6 plays an important role in the secretion of PGE2 in this immunomodulatory effect [105].

Moreover, such as NO, inducible nitric oxide synthase (iNOS)-27, and IL-10 have been related to mediate the MSCs immunosuppressive function [72]. MSCs also suppress the inflammatory responses of natural killer (NK) cells by secreting TGF-β and IL-6 [106]. MSCs also can regulate immune cell function through various cytokines. IFN-γ upregulated IDO expression in MSCs via the JAK-STAT1 signaling pathway, which was involved in inhibiting mononuclear cell proliferation and M2 macrophage polarization [107]. MSCs can inhibit fibrous remodeling and apoptosis, stimulate stem cell proliferation, promote angiogenesis, and decrease oxidative stress through regulating TGF- β, VEGF, ADAMTSs, MMPs, and TIMPs [108].

MSC-bedded media also significantly reduced the production of TNF-α, NO, and PGE2 and the activation of NF-κB. A significant reduction of degranulation, phagocytic activity, and their migratory ability was observed in the presence of the chemokine CCL2. Oxidative stress and mitochondrial dysfunction were inhibited by MSCs-bedded media which also reduced the production of TNFα by M1 macrophages while enhancing TGF-β1 and IL-10 release by M2 macrophages [109]. In addition, when cocultured with MSCs, chondrocytes were able to maintain a stable mature phenotype with decreased expression of hypertrophic and fibrotic markers, which was partly due to the secretion of HGF by the MSCs [17].

MSCs not only reduce tissue damage but also reduce the OA related pain. MSCs downregulated ADAMTS-5 expression but inhibited the expression of anticalcitonin gene related peptide (CGRP) and increased the expression of TNF-α stimulated gene/protein -6 (TSG-6) indicating the suppression of the central sensitization of pain [1,20].

Lots of study results suggest that MSCs secrete many trophic factors that modulate the injured tissue environment. These factors orchestrate subsequent regenerative processes including cell migration, proliferation, differentiation, and extracellular matrix synthesis [110,111,112,113].

### Enhanced MSC Function

The conventional MSCs culturing method on a culture plate does not provide the physiological microenvironment for optimum extracellular vesicle production [114]. Secretome profiles of MSCs are reflective of their local microenvironments. These biologically active factors from secretome exert an impact on the surrounding cells, eliciting regenerative responses.

Nowadays, lots of studies were evaluated for the enhanced efficacy of MSC using culturing platform for therapeutic application. Exposure to TNF-α during in vitro culture, MSCs reveals an increase in migration, proliferation, and the osteogenic capacity [115]. Poly-L-lactide-co-ε-capro- lactone (PLCL) electrospun fiber sheets also enhanced the paracrine signaling of MSCs for cartilage regeneration [114]. Exposures of MSCs to pulsed electromagnetic fields could enhance MSCs paracrine effect and chondrogenesis [116]. In addition, chondrogenic preconditioning of MSCs and mechanical stimulation showed synergic effect for cartilage regeneration [117,118]. However, a study reported that chondrogenic predifferentiation of MSCs before transplantation does not enhance cartilage repair compared to undifferentiated MSCs [119]. We need more preclinical studies about the efficacy of pre-enhancing MSCs during the culture period.

## 5. Exosomes

Exosomes are the small extracellular vesicles (EVs) with a diameter range of 30–150 nm secreted by cells for intercellular communication [44]. MSC exosomes are derived from bone marrow, adipose tissue, synovimal tissue, fetal tissues, and the umbilical cord and embryo. Exosomes formed by the inward budding of endosomal membranes during the maturation of multivesicular bodies [120]. Exosomes are secreted through the fusion of multivesicular endosomes with the cell membrane, while microvesicles (diameter range of 50–5000 nm) are secreted through the forward budding of the plasma membrane [26,121]. Through spectrometry and microarray analysis, exosomes carry a complex cargo of proteins, lipids, and nucleic acids (mRNA and miRNA) and have been reported to promote cartilage repair and regeneration [109,122,123]. The crucial role of MSCs-derived exosomes to cartilage repair have been given exciting attention due to the regulation of cell migration, proliferation, differentiation, and extracellular matrix synthesis by recent preclinical studies [112,124,125].

MSCs-derived exosomes provide a new paradigm for the development of cell-free and ready-to-use therapy for cartilage lesions and OA [39]. These exosomes do not have a nucleus structure; thus, they cannot replicate [44]. According to the latest update statement indicated in the Minimal Information for Studies of Extracellular Vesicles 2018 (MISEV2018) report [126], the terminologies of exosome and ectosome should only be used in an experimental research design with the subcellular origin of the EV subtype. If not, operational terminologies for EV subtypes should be applied, which are based on (i) particle size (based on 200 nm; small EVs smaller than 200 nm, and medium and large EVs larger than 200 nm), (ii) surface markers expression or biochemical recognition (for example; CD81^+^, CD63^+^), and (iii) the origin of cells where the EVs are isolated (for example, hypoxic EVs and apoptotic bodies).

Exosome therapy is now widely accepted as the revolutional therapeutic agents that mediate the many therapeutic efficacies of MSCs. Exosome production is more controllable to cell culture techniques and genetic manipulation, ensuring their cost-effective production. Intralesional injection (e.g., intra-articular injection) of chemosynthetic miRNA is relatively safe and efficient for OA treatment, and MSCs-derived exosomes provided the optimal media to package and transport them [39,127].

### 5.1. Therapeutic Carrier Role of Exosomes

After secreting into the extracellular space, exosomes are transported and deliver their contents to their target cells, resulting in the alteration of gene expression. As a result, physiological and biological function modifications have been induced [44,120]. Most exosomes are rich in proteins and lipids with up to 8000 proteins, and 194 lipids are related to exosomes [44]. In addition, exosomes are known as carriers of mRNAs and miRNAs, for intercellular communication [128].

Since exosomes are secreted by numerous cell sources and the content is strongly associated with the cell origin, each exosome may have variant roles in intercellular communication for numerous physiological effects. For example, platelet secretes exosomes containing prostaglandins which can modulate inflammatory reactions [129]. Exosomes have an increasing popularity as an ideal drug delivery system due to their nonimmunogenic character. Because exosomes are a very heterogeneous type, they can carry different proteins on their surface, which facilitate delivery into the cell through receptor-mediated endocytosis upon interacting with the target cells. [112]. Therefore, exosome-assisted drug delivery systems have been investigated by various researchers to deliver a variety of therapeutic agents to their target cells, including miRNA and recombinant proteins, as well as anti-inflammatory cytokine and chemotherapeutic agents [120].

### 5.2. Promoting Cartilage Repair or Regeneration Using Exosomes

Recently, researchers have focused on utilizing stem-cell-derived exosomes as an innovative therapy due to the ease of accessibility, better stability, and unlimited supply for cartilage regeneration [125]. Exosomes contain various growth factors, cytokine, and miRNAs for cartilage regeneration. The regenerative potential of the exosomes was evaluated, and the presence of more than twenty miRNA types was introduced, which were attributed to inducing positive effects in the control of the joint microenvironment [96]. In a previous in vivo animal study, the osteochondral defects were the complete recovery after applying with MSC-derived exosomes in a rat model. The recovered cartilage and osteochondral bone showed nearly normal chondral structural characteristics, such as hyaline cartilage with normal surface regularity and good marginal attachment to the adjacent cartilage, with well-deposited ECM [110].

Beyond regenerative capacity, exosomes also showed chondroprotective effects. For examples, the treatment of synovial explants with MSC-conditioned media was found that there is the inhibition of the expression of matrix degradative enzymes, including MMP-1, MMP-12, MMP-13, and IL-1β, thus inducing the repair of cartilage tissue [110,127,130]. Another study also pointed toward the character of MSCs to protect chondrocytes via the upregulation of type II collagen production to resynthesize the ECM and decrease the apoptosis via IL-1β downregulation [131,132]. Apart from restoring ECM, the enhanced expression of type II collagen could prevent the hypertrophy of chondrocytes, thus avoiding cartilage degeneration [18,133].

Moreover, exosome’s chondroprotective effect has been demonstrated in several animal models with induced joint disease. This in vivo chondroprotective effect was achieved by increasing the expression of chondrocyte markers (COL2A1 and aggrecan) while suppressing catabolic genes (including ADAMTS-5 and MMP-13) to prolong the survival of degenerative chondrocytes induced with IL-1β [18]. Furthermore, MSCs-derived exosomes potentially assisted in cartilage regeneration procedure by preventing chondrocyte apoptosis through the upregulation of antiapoptotic proteins, including Bcl-2 and surviving [26].

In fact, exosomes are a more effective intercellular communication option, than only proteins or small biochemical molecules such as mRNAs and miRNAs, which can regulate recipient cell gene expression and protein production. The ability of exosomes to deliver proteins and genetic material into cells at a distance accepted them as an ideal candidates for cell-free therapy.

## 6. Exosomal miRNA

The therapeutic potential of MSCs-derived exosomes is usually supported by the presence of biologically suitable miRNA and proteins [134]. More than 1000 proteins have been identified in MSC-derived exosomes, suggesting that the proteome plays a key role in various biological processes including signal transportation, exosome biogenesis, and tissue repair [135,136]. To date, similar to the proteins, MSCs-derived miRNAs have the main potential to modulate cell-to-cell communication. miRNA also influences the progression of various diseases by regulating the signaling pathways of the recipient cells. miRNAs are a group of short, noncoding single-stranded RNAs, with average 19–24 nucleotides, that regulate post-transcriptional gene expression [137]. miRNA is crucial for physiological development and are involved in various biological processes. Hypothetically, each functional miRNA can interact with 200 mRNAs [138]. Previous studies reported that a battery of miRNAs was related to cartilage regeneration [139,140].

MSCs-derived exosomes carry various types of RNAs, including lncRNA, messenger RNA (mRNA), small noncoding RNA (miRNA, small nuclear RNA, and Piwi-interacting RNA), Y-RNA, ribosomal RNA (rRNA), and transfer RNA (tRNA) [141]. Microarray and sequencing studies reported that exosome’s packing and secretion mechanisms are not random. For example, 18S or 28S RNAs or RNAs larger than 500 nucleotides were not detected in MSC-derived exosomes [142]. One pathway in which exosomes can affect the target cell modulation was suggested to be through the transfer of enclosed mRNA. It has been reported that exosomal mRNAs are translatable, leading to specific protein production. However, the physiological significance of mRNA to cellular functions remains unclear. Because mRNAs only contributed to a small proportion of the RNAs enclosed within the exosomes [143].

### Mechanism of miRNA-Mediated Gene Regulation

To date, more than 24500 miRNAs have been found, and there are certainly more to come [144]. miRNAs are small noncoding RNAs that regulate gene expression by binding to specific regions in the 3′UTR of target mRNAs to cause translational repression, mRNA arrest, and unwinding [145]. miRNA-mediated gene regulation is dynamic. miRNAs can regulate the gene expression via multiple pathways by forming RNA effector complexes, such as miRgonaute, miRNP, or miRISC, along with Argonaute, the most important constituent of all miRNPs [146,147]. The main key factor for miRNA target recognition is based on the Watson and Crick sequence pairing to the proximal 5′ activating region (located at nucleotide 2–8) of the miRNA to the corresponding site in the target mRNA which were mostly located in 3′ UTR [148]. Nevertheless, it was also interpreted that a small subset of miRNAs modulates expression by specifically targeting the 5′UTR and/or coding region of some mRNAs [149]. The biological results of miRNA–mRNA interaction can be modified by several factors contributing to a potential target site’s binding strength and repressive effect. As well as the binding position, binding and repression strength, site accessibility, number of target sites, RNA secondary structure, and sequence flanking may also influence the gene regulation potential [150].

Over many years, lots of studies have been performed to evaluate the potential of MSC-derived exosomes for treating OA and to summarize the miRNAs that play a vital role in recovering chondrocyte and maintaining the normal joint condition (Table 1). The location of miRNA expression and function may differ from pathological or homeostatic roles in the joint. miRNA-targeted exosome therapy appears to be a promising therapeutic agent; however, off-the-target effects should be considered due to multiple gene targets of miRNAs.

## 7. Perspectives

Although MSCs-induced immunomodulatory effects for osteoarthritis have shown great potential for DMOAD agents, we need more studies with standardized clinal protocol and evaluation tool. We need more studies to develop the ideal MSCs source, delivery methods, cell dose, and treatment period. In particular, various research methods that can enhance the efficacy and ensure the safety of MSCs therapy should be discussed.

Gradually, there could be a paradigm shift to MSCs-derived exosomes treatment for a more accurate and detailed target and less cell-related risk than MSCs treatment itself [174]. miRNAs transferred by MSC-derived exosomes have been documented as an essential therapeutic agent to suppress cartilage degradation and enhance chondrogenesis. With these positive findings, lots of studies have been carried out to enrich specific miRNAs composition in EVs for more predictable and ideal clinical outcomes [165]. The miRNA enrichment technique could be performed by the cell line overexpressing or by directly loading miRNAs into exosomes using physical or chemical methods.

RNA binding proteins (RBPs) play a key role in sorting and packaging miRNA into EVs. RBPs could be enriched or silenced in stem cells to modulate the miRNA contents in EVs [175]. Among the nine RBPs, the silencing of MVP induced a 50% reduction in total RNA present in EVs, indicating its critical role in the efficiency of miRNA transport into exosomes [176].

Apart from cell transfection, direct delivery of the desired miRNA into EVs is also an efficient and amenable approach to enrich miRNA method. This could be performed by incubating EVs with the selected miRNAs, with or without a calcium chloride (CaCl2) buffer media [177]. CaCl2 enhances miRNA uptake into the EVs by promoting the interactions between miRNAs and the EV surface [178]. Furthermore, the heat-shock method can be used to change the fluidity of the exosomal membrane, which facilitates the entry of miRNA into EV [178]. Electroporation is another technique that facilitates the entry of miRNA [179]. Although the idea sounds plausible, the electroporation method for EVs is still in the infancy steps, and the existing limitations would require further improvement [44].

The exosome delivery method to the OA joints is also under intense study. Studies are ongoing to identify suitable scaffolds or biomaterials for more efficient delivery of the exosomes [44]. The scaffold should protect and preserve the exosomes with biological safety. Encapsulation within the carrier scaffolds also permits the sustained delivery of exosomes for an extended period. It is essential to overcome the exosome’s limitation, which has a short half-life in vivo, and multiple injections might be needed to achieve the desired therapeutic results. In the future, multiple injections can be avoided when persistent drug release is accomplished with the assistance of ideal scaffolds.

**Figure 3:**
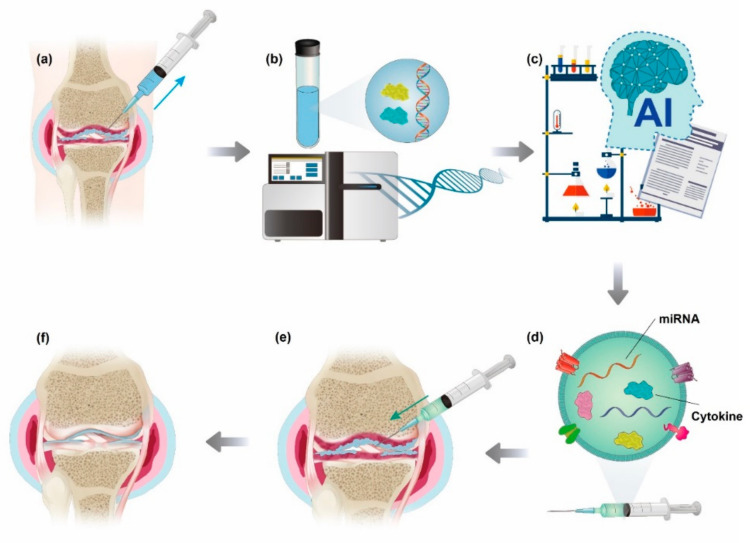
The future paradigm of osteoarthritis (OA) treatment. (**a**,**b**) After analyzing the patient genome and joint synovial fluid, (**c**) we can find crucial factors including proinflammatory cytokine and miRNA for chondral damage of each patient based on big data and artificial intelligence (AI). (**d**) We can arrange a personalized ideal exosome to control the key OA pathway by applying enhancing miRNA and exosome packaging techniques. (**e**) Single or twice injection for local delivery and (**f**) promoting cartilage regeneration and recovery to normal joint conditions.

We need a big database of miRNA and protein profiles in EVs related to cartilage regeneration. This database could help us to better understand the role of miRNA in the treatment and diagnosis of OA [180]. Recent studies suggested that the miRNAs such as miR-9, miR-29, miR-101, miR-181a, and miR-221 and pathways such as Wnt, NF-kB, HIF-1, and PI3K-Akt act as a key role for OA regulation [181,182]. In the future, we can set a personalized OA treatment plan for each patient using big-data and artificial intelligence (AI) deep learning [183]. After analyzing the patient genome and joint synovial fluid, we can find crucial factors including proinflammatory cytokine and miRNA for chondral damage of each patient [184,185]. After that, we can make a personalized ideal exosome for control key OA pathway with the help of AI. By applying enhancing miRNA and exosome packaging techniques, we can obtain the persistent delivery effect by single or twice injection (Figure 3).

## 8. Conclusions

Although MSCs-induced anti-inflammatory and immunomodulatory effects for osteoarthritis have shown great potential in repairing damaged cartilage and joint as DMOAD agents, more studies are needed about safety, mechanism of action, and efficacy. We also need lots of clinical data about injection route, dosage, and treatment interval.

Especially, MSCs-derived exosomes could be game changers for treating cartilage damage and OA. Exosomes contain miRNAs and proteins that can regulate cartilage regeneration by enhancing chondrocyte proliferation, decreasing inflammatory pathways, attenuating apoptosis, promoting chondrogenesis, and increasing cartilage matrix secretion. In addition, enrichment exosome techniques with specific miRNAs have shown promising results in cartilage regeneration in vitro and in vivo studies.

However, we need to keep in mind that although exosome-miRNA-targeted therapy appears to be a promising therapeutic way, off-target effects should be considered due to the multiple targets of miRNA. We still have a long way to go to control OA.

## Figures and Tables

**Table 1:** Cartilage regeneration-related miRNAs from MSC-derived exosomes.

miRNA	Origin	Target	Actions
miR-9-5p	BMSCs	Syndecan-1	has anti-inflammatory and cartilage protective effects on osteoarthritis [151].
miR-22	BMSCs	PPARA/BMP-7	inhibition upregulates BMP-7 and PPARA expression, inhibits IL-1 expression, and suppresses MMP-13 expression in OA chondrocytes [152].
miR-23b	BMSCs	PKA	induces chondrogenic differentiation of human MSCs by inhibiting PKA signaling [153].
miR-23c	BMSCs	FGF2	inhibits articular cartilage damage recovery by regulating MSCs differentiation to chondrocytes via reducing FGF2 [154].
miR-26a-5p	BMSCs	PTGS2	promotes the survival of synovial fibroblasts and reduce synovitis damage [155].
miR-30a	BMSCs	DLL4	promotes chondrogenic differentiation of mesenchymal stem cells through inhibiting DLL4 expression [156].
miR-92a	BMSCs	Noggin3	targets Noggin3 and activates the PI3K/Akt/mTOR pathway to positively regulate the proliferation and matrix synthesis of chondroprogenitors [157].
miR-92a-3p	BMSCs	Wnt5a	regulates cartilage development and homeostasis by targeting Wnt5a [158].
miR-100-5p	IPFP-MSCs	mTOR	inhibits mTOR autophagy signaling pathway to enhance chondrocyte autophagy [159].
miR-124-3p	BMSCs	circHIPK3/MYH9	chondrocyte proliferation and migration induction and in chondrocyte apoptosis inhibition via MYH9 axis [132].
miR-125b	BMSCs	ADAMTS-4	suppresses IL-1-induced upregulation of ADAMTS-4 in human OA chondrocytes [160].
miR-127-3p	BMSCs	Wnt/β-catenin	inhibits CDH11, blocks the Wnt/β-catenin pathway in chondrocytes, and reduces the chondrocyte damage in osteoarthritic joints [161].
miR-129- 5p	SMSCs	HMGB1	declined the inflammatory response and apoptosis of chondrocytes via HMGB1 upregulation [162].
miR-135b	BMSCs	Sp1a MAPK6	promotes chondrocyte proliferation and cartilage regeneration in OA by downregulating Sp1a in chondrocytes [163]
miR-136-5p	BMSCs	ELF3	promotes chondrocyte proliferation and inhibits chondrocyte degeneration [164].
miR-140-5p	SMSCs	Wnt/YAP	enhances ECM secretion and induces proliferation and migration of chondrocytes via activating YAP as well as preventing osteoarthritic joint damage [165].
miR-145	BMSCs ADMSCs	Sox9/MKK	inhibition upregulates Sox9 expression and promotes MSC chondrogenesis [166]. attenuate TNF-α-driven cartilage matrix degradation in osteoarthritis via direct suppression of MKK4 [167].
miR-199b-5p	BMSCs	JAG1	positive regulators to modulate chondrogenic differentiation of C3H10T1/2 cells by targeting JAG1 [168].
miR-210	BMSCs	HIF-3α	promotes chondrocyte proliferation and extracellular matrix deposition [169].
miR-218	SDSCs	HPGD	induces chondrogenic differentiation with regulatory role on 15-hydroxyprostaglandin dehydrogenase (HPGD) [170].
miR-221	BMSCs ADMSCs	MDM2	downregulates MDM2 to prevent slug protein degradation, which negatively regulates chondroprogenitor proliferation [171].
miR-320	BMSCs	MMP-13/ SOX9	downregulates MMP-13 expression / up- regulate SOX9 expression to induce cartilage differentiation [172].
miR-361-5p	BMSCs	DDX20 NF-κB	inhibits the NF-κB signaling pathway via targeting DDX20 [173].
miR-449a	BMSCs	SIRT1	targets SIRT1 and lymphoid enhancer-binding factor-1 (LEF-1), and increased cartilage regeneration and expression of type II collagen [139].

BMSCs: bone marrow stem cells; IPFP-MSCs: infrapatellar fat pad mesenchymal stem cells; SDSCs: synovial membrane derived stem cells; and ADMSCs: adipocyte-derived stem cells.
